# Advancing Quantitative Susceptibility Mapping With 2.5D Diffusion Models for Rapid Intracranial Hemorrhage Quantification

**DOI:** 10.1002/mrm.70358

**Published:** 2026-03-24

**Authors:** Zhuang Xiong, Yang Gao, Feng Liu, Derek Emery, Ken Butcher, Alan H. Wilman, Hongfu Sun

**Affiliations:** ^1^ Image X Institute, Sydney School of Health Sciences, Faculty of Medicine and Health University of Sydney Sydney Australia; ^2^ School of Computer Science and Engineering Central South University Changsha China; ^3^ School of Electrical Engineering and Computer Science University of Queensland Brisbane Australia; ^4^ Department of Biomedical Engineering University of Alberta Edmonton Canada; ^5^ School of Clinical Medicine University of New South Wales Sydney Australia; ^6^ School of Engineering University of Newcastle Newcastle Australia

**Keywords:** diffusion models, echo planar imaging (EPI), intracranial hemorrhage (ICH), QSMDiff, quantitative susceptibility mapping (QSM)

## Abstract

**Purpose:**

To develop a generative diffusion model‐based approach for robust and efficient quantitative susceptibility mapping (QSM) reconstruction in intracranial hemorrhage (ICH), applicable to both standard gradient echo (GRE) and rapid echo planar imaging (EPI) acquisitions.

**Methods:**

QSMDiff, an unsupervised diffusion model for 3D QSM dipole inversion, was proposed. Three volumetric partitioning strategies including 2D slices, 3D patches, and 2.5D slabs were evaluated, and the memory‐efficient 2.5D slab approach was adopted to balance accuracy and efficiency while maintaining anatomical fidelity. A conditional sampling mechanism ensured consistency with measured local fields, and a three‐stage training data‐generation strategy combining public dataset, synthetic QSM, and ICH lesions simulated from in vivo patients was implemented to overcome data scarcity.

**Results:**

QSMDiff achieved the best overall performance in simulation studies, with SSIM of 0.97 ± 0.07, RMSE of 0.04 ± 0.03, and HFEN of 4.49 ± 0.83, demonstrating superior structural fidelity and noise suppression. For in vivo ICH patients scanned with rapid EPI, QSMDiff showed strong agreement with SWI‐QSM references (*R* ^2^ = 0.83), producing susceptibility estimates with minimal bias and variance. Qualitative evaluation confirmed enhanced resolution and effective artifact suppression in conditions of low SNR, limited resolution, and motion.

**Conclusion:**

QSMDiff achieves high‐quality and accurate QSM reconstruction from both standard GRE and rapid EPI scans for ICH assessment. By integrating a 2.5D training strategy with synthetic ICH augmentation, it delivers accurate and reliable susceptibility maps even from lower‐quality acquisitions, offering a practical solution for fast and robust ICH assessment.

## 1 Introduction

Magnetic susceptibility in the human brain is predominantly governed by the presence of hemoglobin, iron, myelin, and calcium. Quantitative susceptibility mapping (QSM) is an established technique [1, 2] for measuring tissue susceptibility from the raw phase signal of magnetic resonance imaging (MRI). QSM has been increasingly recognized as a significant biomarker surrogate and has been used to study neuropathological and neurodegenerative disorders, including Multiple Sclerosis [3, 4], Alzheimer's Disease [5], Parkinson's Disease [6], and Huntington's Disease [7], and to quantify and track the dynamic alterations in cerebral blood oxygenation levels [8, 9] for functional activities [10, 11] or tissue viability [12, 13].

Intracranial hemorrhage (ICH) remains one of the most severe forms of stroke [14, 15]. Accurate assessment of hemorrhage composition, etiology, and evolution is crucial for optimizing treatment and predicting outcomes. During ICH, hemoglobin undergoes progressive biochemical conversion into iron‐rich degradation products, resulting in pronounced susceptibility changes. Conventional T2*‐weighted or susceptibility‐weighted imaging (SWI) depicts these changes only qualitatively as signal loss and cannot reliably distinguish iron‐laden hemorrhage from calcification, since both appear hypointense on magnitude images.

In recent years, QSM has shown promising applications in ICH assessment [16], including distinguishing paramagnetic blood products, such as hemosiderin, from diamagnetic calcifications [17] and monitoring the hemorrhage progression as well as staging the disease [18]. While conventional SWI or gradient echo (GRE)‐based QSM requires 5–6 min of acquisition, such long scans are often not feasible for ICH patients, who are prone to motion and frequently cannot tolerate extended MRI examinations. Echo‐planar imaging (EPI) provides a promising alternative, enabling QSM acquisitions within just a few seconds. However, conventional methods [19, 20, 21, 22, 23, 24, 25, 26] remain vulnerable when applied to fast EPI data, suffering from low signal‐to‐noise ratio (SNR), limited resolution, motion artifacts, and susceptibility distortions [27, 28, 29].

ICH produces large, abrupt susceptibility variations due to concentrated blood products, resulting in strong local field distortions and signal voids. These fundamental differences make QSM reconstruction in ICH uniquely challenging, as standard methods cannot reliably recover susceptibility in and around hemorrhagic regions. End‐to‐end U‐net [30] based deep learning methods [31, 32], such as QSMNet [33], QSMGAN [34], and AFTER‐QSM [35], have been shown to enhance performance with general benefits for ICH. Model‐based [36, 37, 38] and unsupervised approaches [39, 40, 41] enhance generalizability by integrating physics‐based information with deep neural networks. More recently, iQSM [42] and iQSM+ [43] achieve single‐step QSM reconstruction directly from raw phase data. However, none of these deep learning methods have been extensively evaluated on EPI‐based scans on ICH patients.

Recently, generative diffusion models [44, 45], such as Denoising Diffusion Probabilistic Models (DDPMs) [44], have gained attention in medical imaging for their zero‐shot capability in solving inverse problems [46, 47, 48, 49], offering potential for robust QSM reconstruction under the noisy, artifact‐prone conditions of EPI acquisitions in ICH. However, DDPMs are computationally intensive and generally ill‐suited for 3D tasks such as QSM reconstruction. As a consequence, most 3D approaches adopt latent diffusion models (LDMs) [50], which map images into a latent space via a pretrained autoencoder and have been applied to 3D MR image super‐resolution [51], image translation and synthesis [52, 53, 54]. Nevertheless, LDMs require robust pretrained autoencoders and large‐scale training datasets, often unavailable in specialized modalities such as QSM. Other methods have introduced patch‐based 3D Diffusion [55, 56] or slice‐wise 2D Diffusion [57, 58], in an attempt to balance reconstruction quality and efficiency, but this trade‐off remains unresolved in data‐scarce domains.

Recent diffusion‐based QSM methods, including Diffusion‐QSM [59] and ACE‐QSM [60], demonstrate the promise of generative models for dipole inversion. The Diffusion‐QSM [59] method trained a 3D patch‐based unconditional diffusion prior on healthy subjects, which was later combined with the physical constraints for QSM reconstruction from 3D GRE acquisitions in an unsupervised manner. ACE‐QSM trained a conditional diffusion model pairing the measured short‐TE QSM with the desired long‐TE QSM in a supervised manner. A more generalized diffusion‐based framework that remains robust under challenging conditions, such as rapid 2D EPI‐based ICH QSM reconstruction, is still missing.

To overcome these limitations, we propose QSMDiff, a diffusion‐based framework for QSM dipole inversion tailored to rapid EPI acquisitions in ICH. Unlike existing latent diffusion approaches that rely on pretrained autoencoders and large datasets, QSMDiff adapts generative modeling to the constraints of efficiency and data scarcity in specialized modalities such as QSM. By introducing a more efficient training and sampling scheme alongside an iterative augmentation process, QSMDiff enables robust susceptibility mapping across diverse clinical protocols. The method produces visually coherent, high‐quality reconstructions from both motion‐affected 3D GRE sequences and 2D EPI scans of ICH patients.

## 2 Methods

### 2.1 Overview of Diffusion Models

DDPM [44] is a generative model that produces in‐distribution high‐fidelity images by iteratively denoising samples drawn from Gaussian noise through forward and reverse diffusion processes. In the forward diffusion process, Gaussian noise is incrementally added to the data over a sequence of steps, till a complete Gaussian distribution, that is, pure noise $q\left(x_{T}\right)=N\left(x_{T};0,I\right)$ is reached:

(1) $$q\left(x_{t}|x_{t-1}\right)=N\left(x_{t};\sqrt{1-\beta_{t}}x_{t-1},\beta_{t}I\right).$$

q represents the forward process, $x_{t}$ denotes the intermediate status during denoising at time t. $\beta_{t}$ is a time‐dependent variance scalar that controls the amount of noise added at each step. This process is typically modeled as a Markov process, meaning each step only depends on the previous one, leading to an accumulative transformation from the original data:

(2) $$q\left(x_{t}|x_{0}\right)=N\left(x_{t};\sqrt{{\bar{\alpha }}_{t}}x_{0},\left(1-{\bar{\alpha }}_{t}\right)I\right),$$

where $\alpha_{t}=1-\beta_{t}$ and ${\bar{\alpha }}_{t}=\prod_{t=1}^{T}\alpha_{t}$. The reverse process is a learnable step, with a neural network parameterized by θ, where $\sigma_{t}$ is a time‐dependent hyperparameter representing the standard deviation of the added noise at each reverse step. Following the standard DDPM formulation, we set $\sigma_{t}^{2}={\tilde{\beta }}_{t}=\beta_{t}\left(1-{\bar{\alpha }}_{t-1}\right)/1-{\bar{\alpha }}_{t}$. It gradually recovers original images from noise corruption:

(3) $$p_{\theta }\left(x_{t-1}|x_{t}\right)=N\left(x_{t-1};\mu_{\theta }\left(x_{t},t\right),{\sigma_{t}}^{2}I\right).$$

This is achieved through the loss function below of predicting the added noise in each step during the forward process:

(4) $$L=E_{x_{0},t∼[1,T],\epsilon ∼N(0,I)}\left[{\left\|\epsilon -\epsilon_{\theta }(\sqrt{{\bar{\alpha }}_{t}}x_{0}+\sqrt{1-{\bar{\alpha }}_{t}}\epsilon ,t)\right\|}^{2}\right]$$

Once the diffusion model, parameterized by θ, is trained on image data, it can generate high‐quality samples by reversing the diffusion process. Starting from standard Gaussian distribution, the model iteratively applies learned denoising steps to produce coherent images. In the unconditional setting, this procedure relies solely on the learned data distribution, without any external conditioning but based on only previous status:

(5) $$p_{\theta }\left(x_{0:T}\right)=p\left(x_{T}\right)\prod_{t=1}^{T}p_{\theta }\left(x_{t-1}|x_{t}\right).$$

### 2.2 QSMDiff: Solving QSM With Diffusion Models

When placed in an external field $B_{0}$, an object with magnetic susceptibility χ induces its local field inhomogeneity ∆B. This field perturbation results from a spatial convolution between magnetic susceptibility and the unit magnetic dipole D, which can be formulated as a multiplication in the K‐space after Fourier transform (F):

(6) $$\varphi =∆B/B_{0}=F^{-1}DF\chi .$$

In standard QSM reconstruction pipeline, the measured MR phase is first unwrapped and normalized to a frequency shift map, followed by background field removal to isolate the local field perturbation ∆B within the region of interest. The subsequent dipole inversion step then estimates the underlying tissue susceptibility distribution χ from this local field, as described by Equation (6), which is an ill‐posed inverse problem primarily due to the zeros and small values near the cones of the dipole kernel D in k‐space. In practice, phase contributions other than local tissue susceptibility sources, in particular the tissue‐air susceptibility interface, are largely mitigated by the background field removal step [61], with some other phase errors introduced by chemical shift or flow effect neglected in brain QSM.

**FIGURE 1:**
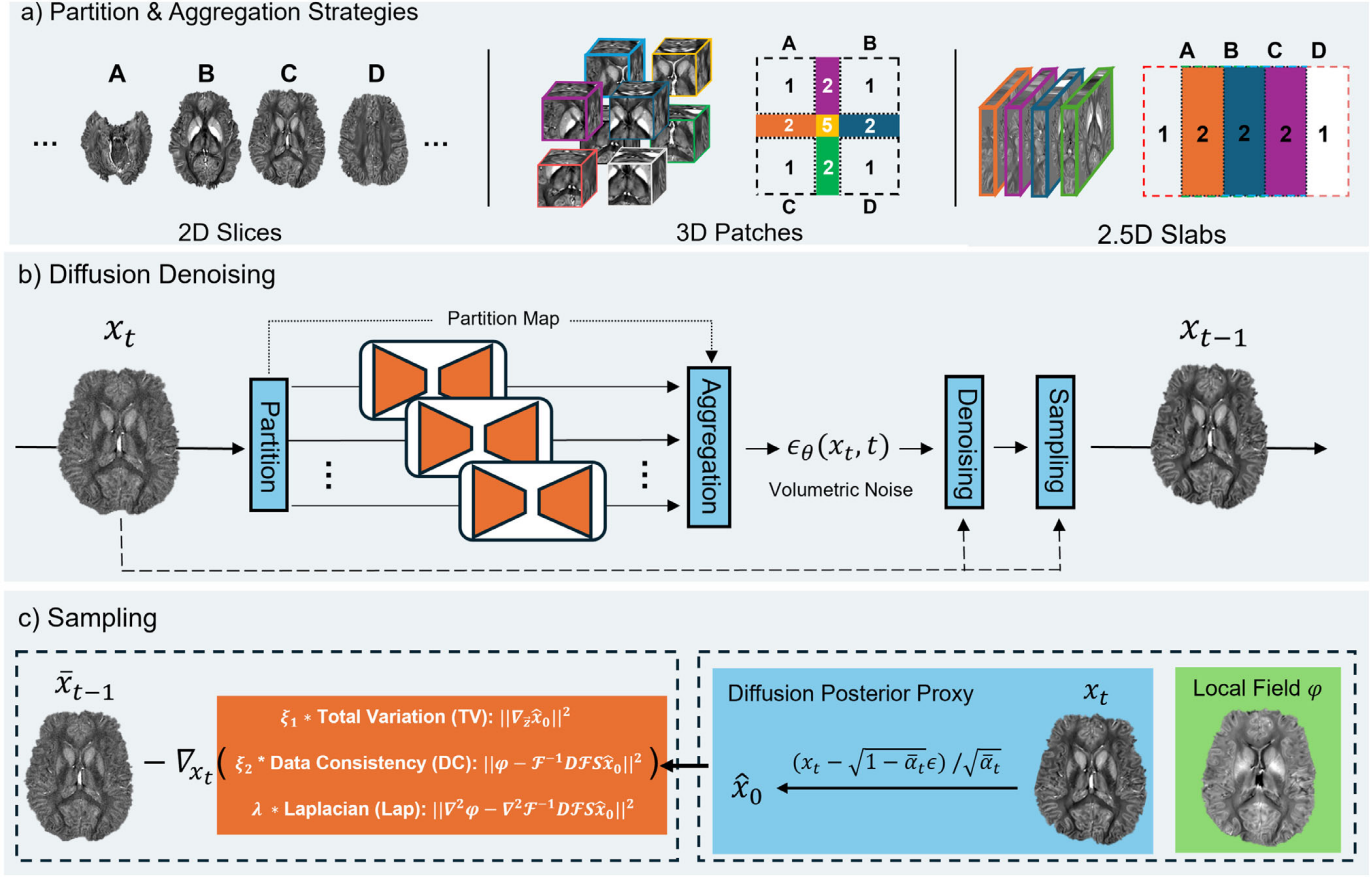
(a) Illustration of different partition and aggregation strategies including 2D slices, 3D patches and 2.5D slabs, together with corresponding overlapping maps for 3D and 2.5D. (b) Illustration of a 2.5D conditional denoising step for QSMDiff. The noisy 3D volume $x_{t}$ is first partitioned into fractions, each of which is fed to diffusion model for denoising in parallel. The predicted noise maps are aggregated and removed. (c) Conditional sampling proxied based on Diffusion Posterior Sampling (DPS), corresponding to the sampling block illustrated in (b).

Our QSMDiff framework introduces an innovative partition‐aggregation approach to compensate for memory limitations during training, enabling an unsupervised diffusion model to serve as a generative prior for QSM. As illustrated in Figure 1a, we evaluate three partitioning and aggregation strategies for QSM reconstruction: 2D slices, 3D patches, and a newly proposed 2.5D slab approach. While 2D and 3D strategies have been previously applied in other medical imaging tasks, they have not been systematically investigated for QSM. In the 2D partition, the volumetric 3D image is decomposed into axial slices. The 3D partition produces overlapping cubic patches, whereas the 2.5D partition generates overlapping slabs along the in‐plane direction, with each slab comprising a stack of consecutive axial slices. For both the 3D and 2.5D strategies, the number of overlaps per voxel is recorded in the corresponding partition maps, as shown in the figure, to facilitate later aggregation. This partition process can be modeled as an operation P:

(7) $${\left\{x_{0}^{i}\right\}}_{i=0\ldots n}=P\left(x_{0},d,o\right),$$

where d and o are the fraction size and the stride in the overlapping mask. These fractions can then be used to train the fractional QSM diffusion models through the standard DDPM forward and reverse processes, as detailed in Algorithm 1. Following training, the fractional diffusion models are tailored to the QSM dipole inversion problem. Reconstruction proceeds via denoising (see Algorithm 2, line 6) and conditional sampling blocks in Figure 1b, beginning with a 3D Gaussian noise volume matched to the subject's full brain dimensions. Through iterative reverse steps, this noisy initialization is progressively refined into a clean susceptibility map $x_{0}$. Each denoising step $x_{t}$ → $x_{t-1}$ integrates both learned priors from the fractional diffusion models and data consistency constraints informed by the measured local field.

Specifically, each of the noisy partitions is fed into the pre‐trained diffusion model to predict the contained noise. These noise predictions from different partitions are then assembled into the full 3D volume by averaging overlapping regions. This average noise is subsequently removed from $x_{t}$ to results in a less noisy full brain 3D volume ${\bar{x}}_{t-1}$.

Diffusion Posterior Sampling (DPS) [62] in Figure 1c is then conducted on ${\bar{x}}_{t-1}$ to enforce data consistency with the whole‐brain local field measurement φ according to Equation (6). As depicted in Algorithm 2, the conditional sampling step in the reverse process can be formulated as:

(8) $$x_{t-1}={\bar{x}}_{t-1}-\xi_{1}\cdot \nabla_{x_{t}}\mathrm{DC}\left(\varphi ,{\hat{x}}_{0}\right)-\xi_{2}\cdot \nabla_{x_{t}}\mathrm{Lap}\left(\varphi ,{\hat{x}}_{0}\right)-\lambda \cdot \nabla_{x_{t}}\mathrm{TV}\left({\hat{x}}_{0}\right),$$

where DC stands for data consistency loss and is defined as:

(9) $$\mathrm{DC}\left(\varphi ,{\hat{x}}_{0}\right)={\left\|\varphi -F^{-1}DF{\hat{x}}_{0}\right\|}^{2},$$

and Lap denotes the second order difference (i.e., Laplacian loss) to preserve the structural boundaries:

(10) $$\mathrm{Lap}\left(\varphi ,{\hat{x}}_{0}\right)={\left\|\nabla^{2}\varphi -\nabla^{2}\left(F^{-1}DF{\hat{x}}_{0}\right)\right\|}^{2},$$

with ${\hat{x}}_{0}$ being the fully denoised QSM prediction from the diffusion models. $\xi_{1}$ and $\xi_{2}$ are the weighting factors that balance the loss terms. TV denotes total variation penalty that preserves image continuity.

ALGORITHM 1 **Training.**

1: **determine** $d,o,\alpha_{t}$

2: ${\left\{x_{0}^{i}\right\}}_{i=0\ldots n}=P\left(x_{0},d,o\right)$

3: **repeat**

4:   t∼{1,…,T}, ϵ∼N(0,I)

5:   ${x_{t}}^{i}\leftarrow \sqrt{{\bar{\alpha }}_{t}}{x_{0}}^{i}+\sqrt{\left(1-{\bar{\alpha }}_{t}\right)}\epsilon$

6:   **gradient step on**:

7:   $\nabla_{\theta }{\left\|\epsilon -\epsilon_{\theta }\left({x_{t}}^{i},t\right)\right\|}^{2}$

8: **until** converged

ALGORITHM 2 **Sampling.**

1: $x_{T}∼N\left(0,I\right)$ **keep** $d,o,\alpha_{t}$; **determine** $\xi_{1},\xi_{2},\lambda$

2: **for** t=T,…,1 **do**

3:   ${\left\{x_{t}^{i}\right\}}_{i=0\ldots n}=P\left(x_{t},d,o\right)$

4:   ${\hat{\epsilon }}_{t}=P^{-1}\left(\epsilon_{\theta }\left({\left\{x_{t}^{i}\right\}}_{i=0\ldots n},t\right),d,o\right)$

5:   z∼N(0,I)ift>1else0

6:   ${\bar{x}}_{t-1}=\frac{1}{\sqrt{\alpha_{t}}}\left(x_{t}-\frac{1-\alpha_{t}}{\sqrt{\left(1-{\bar{\alpha }}_{t}\right)}}{\hat{\epsilon }}_{t}\right)+\sigma_{t}z$

7:   ${\hat{x}}_{0}=\frac{1}{\sqrt{{\bar{\alpha }}_{t}}}\left(x_{t}-\sqrt{1-{\bar{\alpha }}_{t}}\epsilon \right)$

8:   $x_{t-1}={\bar{x}}_{t-1}-\xi_{1}\cdot \nabla_{x_{t}}\mathrm{DC}\left(\varphi ,{\hat{x}}_{0}\right)-\xi_{2}\cdot \nabla_{x_{t}}\mathrm{Lap}\left(\varphi ,{\hat{x}}_{0}\right)-\lambda \cdot \nabla_{x_{t}}{\mathrm{TV}}_{\vec{z}}\left({\hat{x}}_{0}\right)$

9: **end for**

10:  **return** ${\hat{x}}_{0}$

### 2.3 Training Data Preparation

**FIGURE 2:**
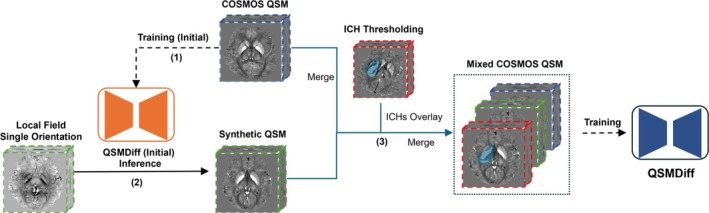
Data preparation for QSMDiff training (2.5D as a representation) containing 3 portions: (1) 2.5D COSMOS axial slabs used for initial training; (2) reconstructed QSM images of healthy brains by the initial QSMDiff model; (3) ICH‐QSM slabs with ICH lesions extracted from patients and overlaid on healthy brain QSM slabs from (1) and (2). All three portions are merged for final QSMDiff model training.

As illustrated in Figure 2, the data preparation for QSMDiff training follows a three‐stage process. First, 20 COSMOS subjects from two publicly available datasets [38, 63], with an isotropic image resolution of 1 mm, were used to train the initial QSMDiff model. This stage also included a comparison of different partitioning strategies, and the details of their performance are presented in Section 3.

Second, this initial QSMDiff model performed a continual data generation that reconstructed 96 healthy subjects of standard single‐orientation ME‐GRE acquisitions (acquisition parameters and QSM pre‐processing steps are detailed in [64, 65]), according to Algorithm 2: Sampling.

Third, 17 ICH patients acquired with SWI sequence were reconstructed using the iQSM+ [43] method and the ICH lesions were segmented by simple threshold (i.e., susceptibility values larger than 0.3 ppm) and randomly overlaid on healthy QSM from the first two stages, producing diverse ICH training samples. It is important to note that the 2.5D diffusion model was trained exclusively on the GRE dataset. The EPI dataset was strictly reserved for testing and evaluation, and was never used during the training phase.

All three datasets were merged to re‐train the final QSMDiff models of three versions: QSMDiff‐2D, QSMDiff‐3D, and QSMDiff‐2.5D.

### 2.4 Training and Hyper‐Parameter Setting

The code implementation of QSMDiff was based on the Ablated Diffusion Model [66] with a modified 3D Unet structure [30]. The Unet architecture has four encoding and four decoding layers, each comprising two residual convolutional blocks. Additionally, an attention layer is incorporated at both the final and initial layers of the encoder and decoder, respectively. The initial number of channels is set to 128, with a predefined channel number expansion factor of [1, 2, 4] applied after each encoder layer, and a reversed order reduction factor implemented in the decoder. Denoising Diffusion Implicit Models (DDIM) sampling [67] with 200 steps was adopted to speed up the reverse process. All work was developed using Pytorch 2.0. QSMDiff was trained for 11 days using one Nvidia H100 GPU with 80 GB vRAM, and the reconstruction was conducted using an RTX 4090 GPU with 24 GB vRAM. The training loss converged after 120 epochs with a batch size of 2. Trilinear interpolation was employed here as the resolution resampling operator *S*. To enhance computational efficiency, the thickness and overlap stride were experimentally set to 16 and 8 for 3D and 2.5D partitioning. We empirically tuned the weight factors $\xi_{1}$ and $\xi_{2}$ to be 1.5 and 4, respectively, which demonstrated on average the optimal performance in different scenarios.

We also trained an alternative version of the QSMDiff model using only healthy brain QSM samples. The effect of including ICH samples is analyzed and demonstrated on two in vivo subjects in Section 3.

### 2.5 Simulation and In Vivo Experiments

To validate the efficacy of the proposed QSMDiff method, comprehensive experiments were conducted, including an ablation study, simulated data analysis, and in vivo experiments on both healthy and ICH human brains. This study was approved by the local ethics board. Both qualitative and quantitative comparisons were performed as follows:

- In vivo Healthy Subject #1: A healthy in vivo brain was scanned on a 3 T Discovery 750 GE system using a 12‐channel head coil. This dataset was used in the ablation study to compare different patching strategies (2D, 3D, and 2.5D). Full brain 3D ME‐GRE data were acquired in a pure‐axial orientation with the following parameters: 8 unipolar readout echoes, first TE = 3.4 ms, echo spacing = 3.5 ms, TR = 29.8 ms, flip angle = 20°, voxel size = 1 mm isotropic, FOV = 256 × 256 × 128 mm^3^, ASSET acceleration factor = 2, and a total scan time of 5.9 min.
- Simulated Healthy Subjects: Quantitative assessment for the ablation experiments was performed using the Structural Similarity Index Measure (SSIM) and the High‐Frequency Error Norm (HFEN) to evaluate structural fidelity and high‐frequency artifact suppression on five simulated healthy subjects. Noise variation was introduced by adding Gaussian noise with progressively increasing standard deviation proportional to the local field average magnitude.
- Simulated ICH Subjects: ICH‐QSM volumes were generated by embedding different ICH lesion patterns into 6 healthy brain QSM volumes randomly drawn from high‐quality COSMOS data. These simulated ICH brains were used for qualitative and quantitative comparison of ICH reconstruction performance.
- In vivo Healthy Subject #2: This subject from a published dataset [68] was scanned using a single‐shot 2D EPI sequence at 3 T with TE = 35 ms, TR = 7.1 s, and imaging resolution of 1 mm isotropic. This dataset was used to evaluate method performance under low‐SNR conditions.
- ICH Patient Cohort: A total of 20 ICH patients were scanned using both a standard SWI sequence and a paired single‐shot 2D EPI sequence on a 3 T Siemens Prisma scanner. The sequence parameters are described in a previous study [27]. Subjects #3, #4, and #5 are shown as representative examples in Section 3, along with quantitative analysis across the full cohort.

For all in vivo experiments, the phase images were unwrapped using the 3D best‐path algorithm [69], and background‐field removal was performed with the RESHARP method [61]. For all simulation experiments, the local field maps were simulated using the source‐to‐field equation (Equation 6) and were used as inputs to ensure controlled evaluation conditions without phase unwrapping or background‐field removal errors.

QSMDiff was compared to one conventional non‐deep learning method (iLSQR [25]) and four deep‐learning‐based QSM methods (Unet [30], AFTER‐QSM [35], iQSM+ [43], and LPCNN [38]) in both simulation and in vivo experiments. To ensure fair comparisons, all in vivo acquisitions were rescaled to 1 mm isotropic resolution and reoriented to pure‐axial slices to meet the requirements of certain deep learning models. Unet and AFTER‐QSM were trained from scratch using the same dataset as QSMDiff, including synthesized ICHs, with paired QSM and local field maps generated via the susceptibility‐to‐field forward model (Equation 6) for supervised learning. In contrast, iQSM+ and LPCNN were evaluated using pre‐trained models provided by their original authors. Similarly, the original iQSM+ model had been trained with additional synthetic ICH patterns, while LPCNN, designed for multi‐orientation in vivo datasets, was only trained on healthy brains and could not be adapted to our synthetic‐ICH training setup. The measured susceptibility values were referenced to the whole brain by setting the mean susceptibility value across the whole brain to zero.

## 3 Results

### 3.1 Ablation Study on Partition and Aggregation Strategies

The ablation study evaluates each partitioning method's performance on QSMDiff with respect to structural detail preservation and image fidelity using Subject #1. Specifically, the 2D, 3D, and 2.5D QSMDiff models were trained on QSM partitions of size 192 × 192, 64 × 64 × 64, and 192 × 192 × 16, respectively. The reconstruction time for Subject #1, with a matrix size of 192 × 192 × 128, was 9, 21, and 15 min for 2D, 3D, and 2.5D, respectively.

**FIGURE 3:**
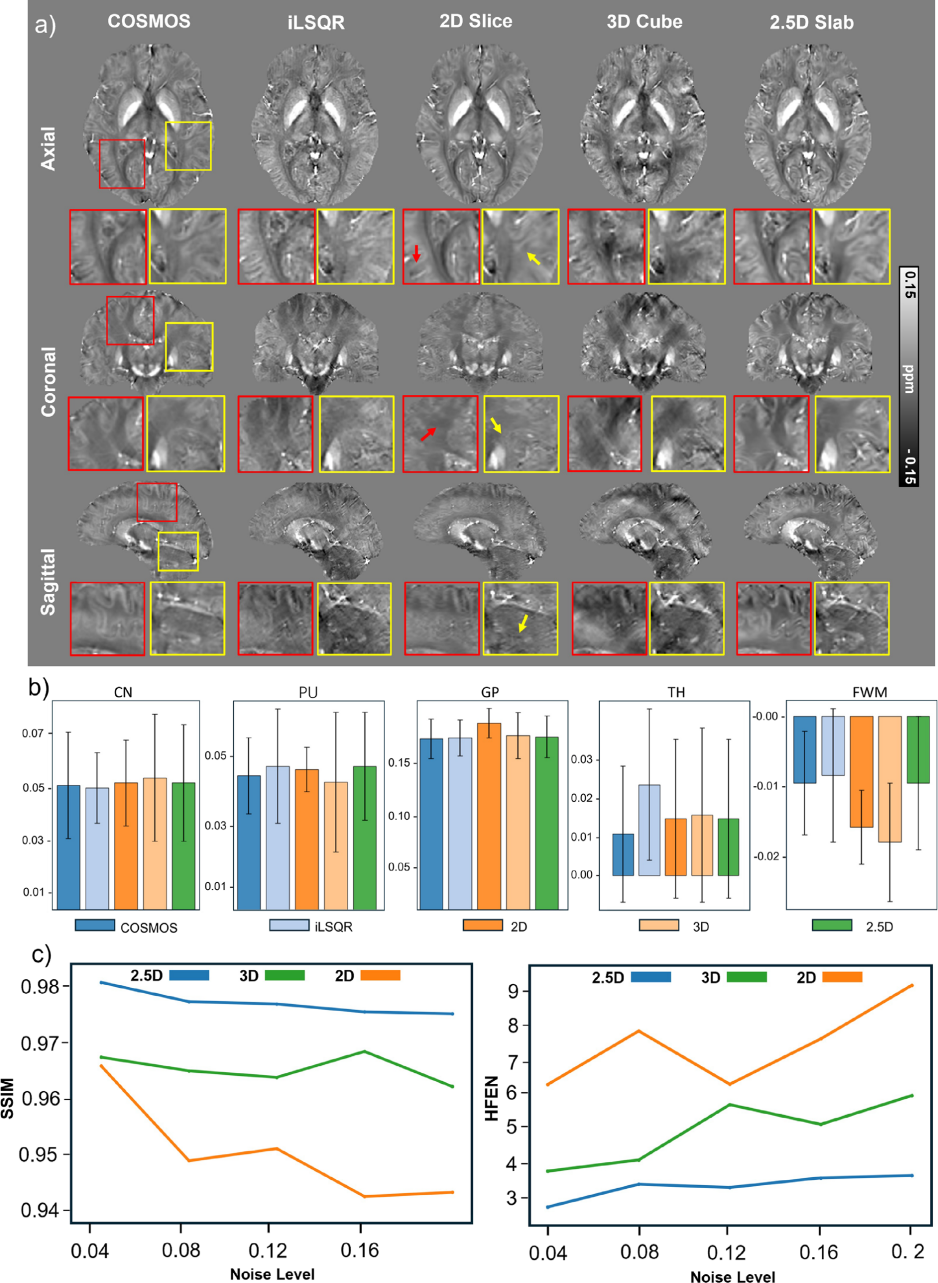
(a) Ablation study from the in vivo Subject #1, comparing different approaches of partition training and sampling for QSMDiff. Arrows from the 2D slice approach are pointing at inconsistent structure due to model hallucination. (b) analysis on 5 ROIs from (a), including Caudate Nucleus (CN), Putamen (PU), Globus Pallidus (GP), Thalamus (TH), and Front White Matter (FWM), selected from the 2D axial slice. (c) Quantitative evaluation of SSIM (left) and HFEN (right) for 2D‐, 2.5D‐, and 3D‐QSMDiff across the simulated subject under five varying noise levels. Each index corresponds to a specific noise level, defined as the standard deviation expressed as a proportion of the regular scale of local field magnitude. Metrics are presented as mean values with standard deviation error bars.

As shown in Figure 3a, the 2.5D approach demonstrated superior performance, producing cleaner reconstructions with reduced noise compared to the reference iLSQR method. It also substantially outperformed the 2D and 3D approaches in preserving accurate structural details and suppressing artifacts, with COSMOS serving as the reference. In contrast, the 2D slice method resulted in noticeable over‐smoothing and a loss of data consistency, as indicated by the arrows in the zoomed‐in sagittal and coronal views. The 3D cube approach preserved more detailed image structures than the 2D method but introduced pronounced shadowing and streaking artifacts.

Quantitative susceptibility values were extracted from five representative brain regions: the caudate nucleus (CN), putamen (PU), globus pallidus (GP), thalamus (TH), and frontal white matter (FWM). As shown in Figure 3b, the 2.5D model shows the closest agreement with COSMOS across all regions, with less than 4.5% of deviations, indicating the best overall quantitative fidelity among the ablated configurations.

The quantitative comparisons on a simulated brain with 5 different noise levels in Figure 3c further demonstrated that the 2.5D method consistently outperformed the other variants, achieving higher SSIM and lower HFEN values across all noise levels. These results indicate that the 2.5D configuration provides the most robust reconstruction under varying noise conditions.

### 3.2 Simulated ICH Brains

We conducted a quantitative analysis of the six brain phantoms with different ICH patterns with results presented in Table 1. In this simulation study, QSMDiff achieved the best overall performance, with an SSIM of 0.97 ± 0.07, RMSE of 0.04 ± 0.03, and HFEN of 4.49 ± 0.83. The local field maps were simulated using Equation (6), corresponding to the QSM forward model applied to COSMOS‐derived susceptibility maps, onto which in vivo human ICH lesion patterns were overlaid. This setup enabled controlled quantitative evaluation on the dipole inversion step, demonstrating that QSMDiff provides superior structural fidelity and noise suppression relative to other methods.

**TABLE 1:** Quantitative evaluation of different QSM methods using six simulated ICH brains.

Metrics	iLSQR	Unet	LPCNN	AFTER‐QSM	iQSM+	QSMDiff
SSIM↑	0.95 ± 0.05	0.95 ± 0.12	0.96 ± 0.12	0.94 ± 0.14	0.97 ± 0.11	**0.97 ± 0.07**
RMSE↓	0.04 ± 0.12	0.07 ± 0.22	0.05 ± 0.22	0.06 ± 0.25	0.04 ± 0.02	**0.04 ± 0.03**
HFEN↓	4.58 ± 0.16	7.36 ± 0.12	5.75 ± 1.26	7.81 ± 0.16	5.11 ± 0.87	**4.49 ± 0.83**

*Note*: ↑ indicates that a higher value reflects better performance (as in SSIM), and ↓ indicates that a lower value reflects better performance (as in RMSE and HFEN). BOLD indicates the best results among all methods.

**FIGURE 4:**
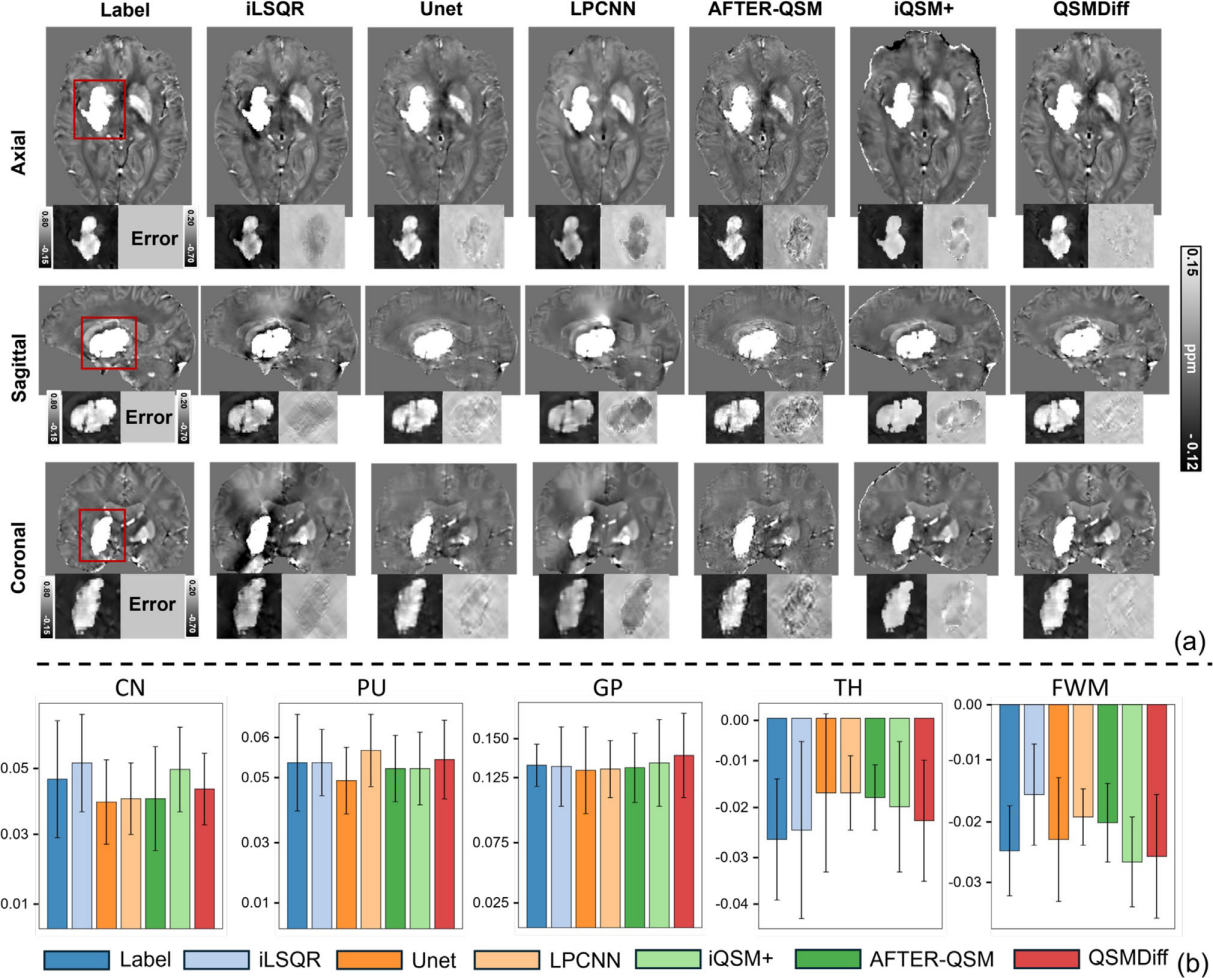
(a) Methods comparison on a simulated COSMOS brain with an in vivo ICH lesion overlayed. Localized regions containing ICH (red boxes) and their error maps were highlighted underneath the images. (b) Susceptibility values were compared across six reconstruction methods. Regions of interest (ROIs) included the caudate nucleus (CN), putamen (PU), globus pallidus (GP), thalamus (TH), and frontal white matter (FWM). The ROIs were drawn in 2D from the corresponding axial slice.

One of the simulated ICH‐QSM reconstruction results is also qualitatively compared in Figure 4, where QSMDiff, iQSM+, and Unet achieved the best visually appealing results with the least shadowing and streaking artifacts in the ICH vicinities, followed by AFTER‐QSM, LPCNN, and lastly the iLSQR method with observable streaking artifacts projecting from ICH regions in sagittal and coronal planes, due to strong susceptibility differences between ICH and surrounding tissue. Moreover, QSMDiff achieved the highest accuracy of ICH measurement, as reported in Table 2 (Subject Sim), which is also evident in the zoomed‐in ROI and the error maps in Figure 4a.

**TABLE 2:** Susceptibility measurements (ppm) of ICH regions obtained from 2D ROIs drawn on three orthogonal views (axial, coronal, and sagittal) corresponding to the largest cross‐sectional area of the lesion, from the simulation subject (Sim) in Figure 4 and in vivo Subjects #3 and #4 in Figure 6. **BOLD** indicates the best results among all methods.

		Label	iLSQR	Unet	LPCNN	AFTER‐QSM	iQSM+	QSMDiff (w/o ICH)	QSMDiff (proposed)
Sim	Axial	0.70 ± 0.11	0.51 ± 0.13	0.66 ± 0.12	0.47 ± 0.10	0.67 ± 0.14	0.65 ± 0.04	0.41 ± 0.16	**0.72 ± 0.10**
	Coronal	0.70 ± 0.11	0.53 ± 0.12	0.63 ± 0.22	0.47 ± 0.16	0.63 ± 0.18	0.64 ± 0.06	0.45 ± 0.18	**0.72 ± 0.11**
	Sagittal	0.76 ± 0.12	0.58 ± 0.15	0.67 ± 0.12	0.46 ± 0.10	0.66 ± 0.14	0.65 ± 0.05	0.56 ± 0.16	**0.76 ± 0.13**
Sub #3	Axial	N/A	1.23 ± 0.31	1.42 ± 0.23	0.91 ± 0.23	1.53 ± 0.31	1.52 ± 0.19	1.34 ± 0.18	1.52 ± 0.23
	Coronal	N/A	1.12 ± 0.32	1.30 ± 0.37	0.89 ± 0.25	1.34 ± 0.51	1.47 ± 0.13	1.39 ± 0.12	1.40 ± 0.28
	Sagittal	N/A	1.24 ± 0.26	1.47 ± 0.18	0.90 ± 0.28	1.48 ± 0.28	1.50 ± 0.05	1.26 ± 0.31	1.54 ± 0.15
Sub #4	Axial	N/A	0.66 ± 0.26	0.61 ± 0.10	0.42 ± 0.19	0.64 ± 0.17	0.78 ± 0.25	0.76 ± 0.32	0.83 ± 0.24
	Coronal	N/A	0.65 ± 0.15	0.52 ± 0.10	0.44 ± 0.13	0.56 ± 0.14	0.77 ± 0.20	0.69 ± 0.26	0.83 ± 0.15
	Sagittal	N/A	0.76 ± 0.15	0.63 ± 0.09	0.46 ± 0.14	0.68 ± 0.111	0.87 ± 0.14	0.87 ± 0.33	0.91 ± 0.12

The ROI analysis of deep gray matter and frontal white matter presented in Figure 4b shows that most of the methods produce consistent measurements in non‐ICH regions, particularly within iron‐rich structures such as globus pallidus and putamen. Among these, QSMDiff, iLSQR, and iQSM+ exhibit the closest agreement with the ground‐truth labels across most ROIs, followed by AFTER‐QSM, LPCNN, and Unet. Standard deviations were comparable across methods, indicating similar measurement precision.

To further evaluate the generalizability of QSMDiff, we conducted an additional simulation study that included both oblique and pure‐axial acquisitions (Figure S1), as well as reconstructions from varying low‐resolution inputs (Figure S2). As shown in Figure S1, QSMDiff demonstrated consistent susceptibility estimation across different slice orientations, maintaining performance parity with the physics‐based iLSQR method regardless of acquisition tilt. Furthermore, results in Figure S2 indicate that QSMDiff is capable of significantly refining blurry anatomical boundaries when processing low‐resolution measurements, effectively sharpening structural definitions where traditional linear methods produced overly smoothed results.

### 3.3 In Vivo Healthy Subject

**FIGURE 5:**
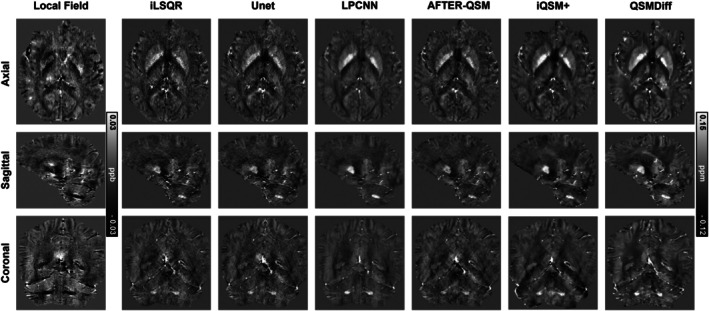
Comparison of QSMDiff with one iterative method (iLSQR), and four deep learning methods (Unet, LPCNN, AFTER‐QSM, and iQSM+) on low SNR data from Subject #2 acquired with a 2D EPI sequence.

In this experiment, we assessed the performance of QSMDiff in handling low SNR data from a 2D single‐shot EPI sequence scanned on the healthy Subject #2. As depicted in Figure 5, QSMDiff achieved visually the best result with substantially improved image quality compared to the other approaches in terms of noise removal. In contrast, alternative methods exhibited notable limitations: high noise levels (e.g., iLSQR, Unet, and AFTER‐QSM), over‐smoothness (iQSM+) or suppressed susceptibility contrast (e.g., LPCNN), resulting in lower‐quality susceptibility maps.

### 3.4 In Vivo ICH Patients

**FIGURE 6:**
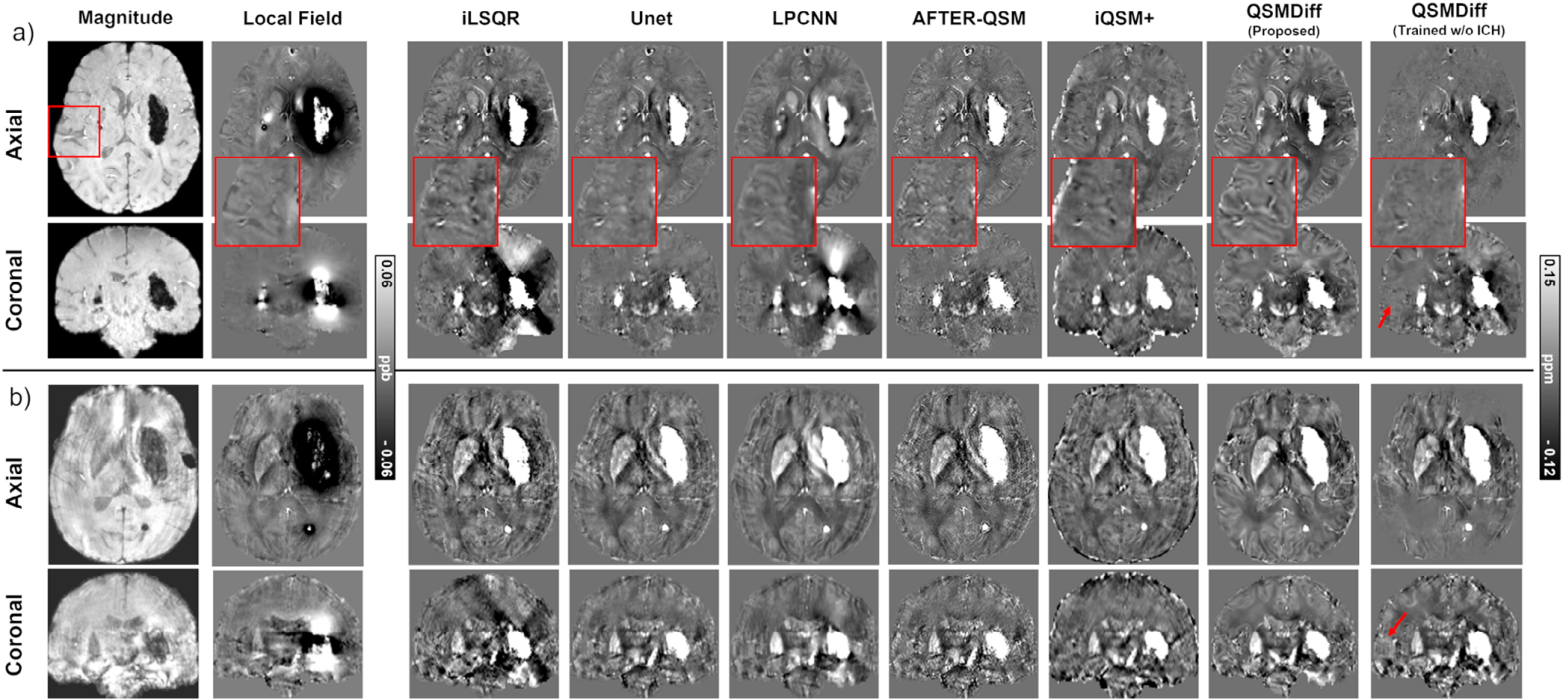
Methods comparison on two in vivo ICH brains, (a) Sub#3 and (b) Sub#4, acquired with a standard SWI sequence. Red arrows in (b) highlight regions where QSMDiff models trained without ICH data failed to produce accurate reconstructions.

Different QSM methods were evaluated on two in vivo ICH patients acquired from SWI scans, namely Subjects #3 and #4, where severe ICH pathologies were exhibited. As shown in Figure 6a, the proposed QSMDiff model (second‐last column) provided visually the best overall tissue contrast with substantially reduced noise levels for both subjects. A zoom‐in inset highlights a region where QSMDiff demonstrates effective contrast enhancement compared with other methods. iQSM+ overall exhibited strong suppression of streaking artifacts around the ICH regions, closely followed by QSMDiff, as evident in both axial and coronal planes. AFTER‐QSM and U‐Net produced smoother but lower‐contrast reconstructions that were largely free of streaking artifacts. In contrast, iLSQR and LPCNN showed pronounced streaking and residual motion artifacts, particularly in regions indicated by the red arrows. Consistently, the proposed QSMDiff achieved the cleanest reconstruction with the least artifacts in Figure 6b compared to the other methods, effectively restoring structural details and image contrast in the brain edge regions. An additional ablation study comparing the 2D, 2.5D, and 3D partition–aggregation strategies using an in vivo brain dataset is provided in Figure S3, complementing the ablation experiments shown in Figure 6. The adopted 2.5D approach ensured the balanced anatomical detail preservation and artifact suppression compared to the 2D and 3D variants.

To assess the effect of ICH augmentation, an additional experiment was conducted on the two in vivo ICH subjects, comparing QSMDiff models trained with (Proposed) and without the inclusion of simulated ICH data. The version trained without ICH augmentation (last column in Figure 6) exhibited markedly reduced performance in generating accurate images for slabs containing hemorrhages; however, it performed similarly to the proposed QSMDiff model on axial slabs that did not contain ICHs.

As summarized in Table 2, quantitative measurements of ICH susceptibilities for Subjects #3 and #4 showed that QSMDiff (proposed), iQSM+, AFTER‐QSM, and Unet produced consistent and relatively higher susceptibility values than iLSQR and LPCNN, following the trends observed in the simulation results. However, the alternative QSMDiff model trained without ICH augmentation tended to underestimate susceptibility values compared to the proposed QSMDiff model. These findings highlight the benefit of incorporating ICH‐specific data augmentation in improving the stability and quantitative performance of QSM reconstruction in patient scans.

**FIGURE 7:**
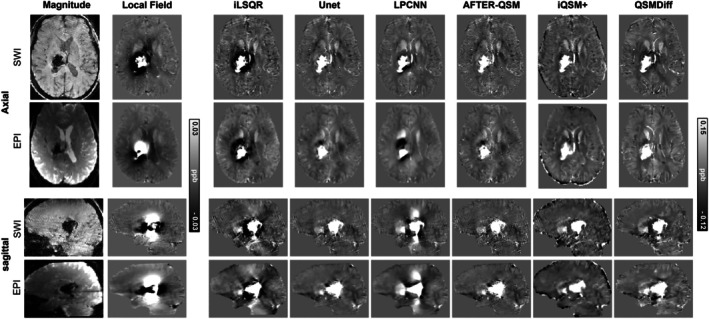
Visual comparisons of ICH‐QSM from various methods on SWI and EPI sequences in axial and sagittal views.

To evaluate performance of EPI‐QSM reconstruction methods on ICH patients, we compared results from Subject #5, scanned with both 1 mm isotropic SWI sequence (5 min) and a rapid 1.5 mm isotropic single‐shot EPI sequence (27 s), in Figure 7. Magnitude images and local field maps were displayed in the first two columns. For the long SWI scan, mild motion can be observed, which represents a common clinical scenario in ICH patients. QSMDiff effectively suppressed motion‐induced artifacts and recovered cleaner structural details on both axial and sagittal views. For the rapid single‐shot 2D EPI acquisitions with a lower resolution, QSMDiff achieved substantial image deblurring and contrast enhancement over the other methods and notably suppressed the streaking and shadowing artifacts. Moreover, QSMDiff maintained the highest visual consistency between SWI and EPI QSM results in both normal tissue and ICH regions.

**FIGURE 8:**
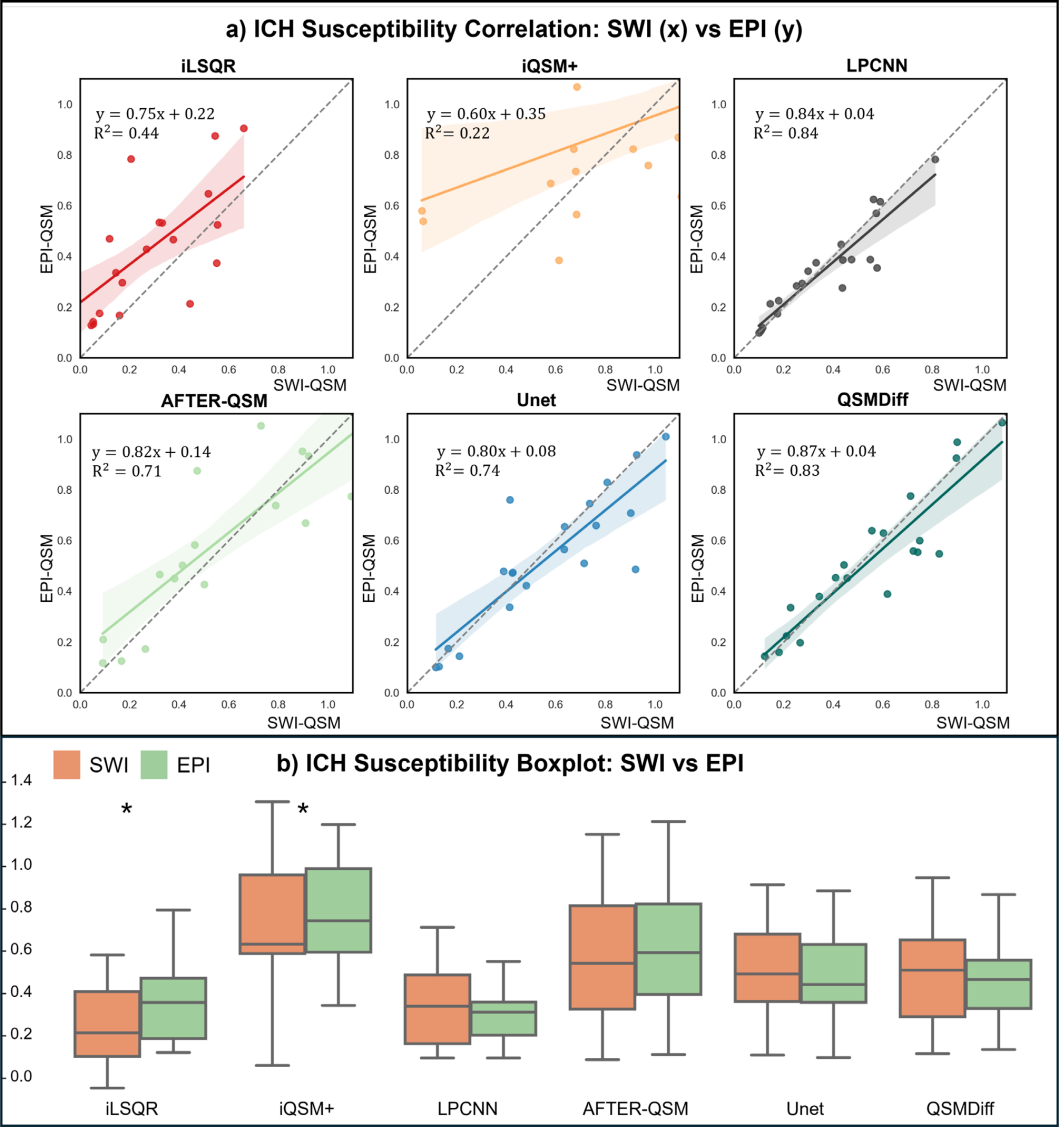
Comparison of ICH susceptibility estimations between SWI‐QSM and EPI‐QSM from different dipole inversion methods on 20 ICH patients. (a) Linear regressions of ICH susceptibilities measured from SWI and EPI with equations and correlation coefficients in the plots. (b) Boxplots comparing the SWI and EPI group means for each method, with asterisks (*) indicating significant group differences.

Quantitative correlation analysis and boxplot group comparisons were conducted on 20 ICH patients to further assess the consistencies of ICH quantification between SWI and EPI of each method. For this analysis, the ICH regions were defined as 2D ROIs on axial slices showing the largest delineated hemorrhage areas, ensuring consistent comparison across methods. The analysis of correlation plots in Figure 8a revealed the comparative performance of various dipole inversion methods in terms of susceptibility correlation between SWI‐QSM and EPI‐QSM for ICH regions. QSMDiff and LPCNN achieved the best results, with linear regression slopes of 0.87 and 0.84, and *R* ^2^ values of 0.83 and 0.84, respectively, indicating strong agreement between the two sequences. By contrast, Unet and AFTER‐QSM demonstrated only moderate correlations (*R* ^2^ = 0.74 and 0.71), while iLSQR and iQSM+ yielded poor correlations (*R* ^2^ = 0.44 and 0.22).

The boxplots in Figure 8b compare group‐level ICH susceptibility values obtained from SWI‐QSM and EPI‐QSM across various reconstruction methods. Most methods demonstrated closely aligned median values and interquartile ranges between SWI and EPI data, indicating consistent susceptibility estimation across the two sequences. However, iLSQR and iQSM+ (marked by asterisks) exhibited significant discrepancies, as confirmed by paired *t*‐tests, with SWI‐derived ICH susceptibilities being lower than those from EPI.

## 4 Discussion

This study introduces QSMDiff, an unsupervised diffusion‐model‐based framework for robust QSM reconstruction. The method was tested on both 3D multi‐echo GRE data and rapid 2D single‐shot EPI acquisitions to assess its adaptability to motion‐prone or low‐SNR imaging conditions in ICH patients. QSMDiff was systematically evaluated against representative QSM reconstruction methods to demonstrate its advantages. iLSQR serves as a widely adopted non‐deep learning traditional baseline, while Unet and AFTER‐QSM are supervised deep learning models designed for end‐to‐end QSM dipole inversion. LPCNN integrates the QSM forward model with deep learning, making it a physics‐informed hybrid approach. While it shares conceptual similarities with QSMDiff, LPCNN remains a supervised framework, unlike QSMDiff's unsupervised generative design. Retraining LPCNN on in vivo ICH data could improve its accuracy, but this is outside the scope of our study. iQSM+ directly processes raw MRI phase data, bypassing phase unwrapping and background field removal, making it more resilient to intermediate errors and effective in handling ICH‐related artifacts.

The implementation of DDPMs for QSM introduces unique computational and methodological challenges. To address these, QSMDiff employs a novel 2.5D patch‐based training and sampling strategy, which reduces memory consumption and computational cost while maintaining reconstruction quality. In contrast, purely 2D patching reconstructs slices independently, provides limited continuity along the through‐plane direction, and relies heavily on total variation regularization, which often results in excessive smoothing. 3D patching processes small, fragmented cubes, thereby limiting contextual awareness and elevating the likelihood of hallucinated structures and artifacts, especially in anatomically heterogeneous areas. To mitigate this, stronger data‐fidelity weighting is needed, which can amplify overfitting effects. The 2.5D approach strikes a balance by employing overlapping axial slabs, preserving spatial consistency while leveraging full axial ROIs for improved contextual awareness. This results in enhanced robustness and finer detail preservation, albeit with higher computational demands.

Generative models, including diffusion models, are fundamentally designed to model their training data distribution and therefore the diversity of the training samples can substantially influence the synthesized image features. For instance, a diffusion model trained solely on healthy datasets is unlikely to generate images of pathological cases. In our experiments, models trained without exposure to ICH brains underestimated ICH susceptibility and failed to capture complex structural features. To broaden our QSMDiff's capability to reconstruct ICH‐QSM, we proposed a three‐stage training strategy, starting with an initial training on healthy subjects and ending with fine‐tuning with ICH patient data. This approach enabled QSMDiff to generalize to pathological cases, achieving accurate ICH susceptibility quantification while reducing artifacts and improving image resolution and SNR.

Our QSMDiff method has certain limitations. The selection of slab thickness in 2.5D training is a factor that influences both computational efficiency and model performance. Ideally, simulation studies using synthetic datasets with known ground truth would allow for precise evaluation of trade‐offs and guide optimal slab configuration. However, such comprehensive study is beyond the current scope of our work due to computational limitations. We selected a slab thickness of 16 slices based on practical constraints, which enabled training with a batch size greater than one (benefits batch normalization), aligned well with common Unet configurations, and remained feasible on personal desktop GPUs with ∼15 GB memory for inference. While other thicknesses could be evaluated, they were either computationally prohibitive or suboptimal due to overlap limitations.

Moreover, hallucination empirically occurs in cases of poor‐quality inputs, particularly when reconstructing from low‐resolution or artifact‐contaminated data, where the generative prior may overcompensate for missing structural details. It may also arise when the model encounters unseen features or pathologies not represented in the training data, such as large brain tumors or calcified regions, leading to anatomically inconsistent or spurious reconstructions. A practical approach to identifying and mitigating hallucination effects is to perform multiple QSMDiff reconstructions with varying prior weightings. Reconstructions using stronger generative prior typically provide optimal image quality, whereas those with lighter prior reduce the likelihood of hallucination but may exhibit slight performance degradation. Comparing reconstructions across different prior strengths can help identify potential hallucination or measurement artifacts, thereby enhancing interpretability and reliability in clinical applications. In addition, recent developments in diffusion‐based reconstruction have introduced approaches for estimating uncertainty maps [47, 70], which can further aid in assessing the reliability and confidence of the reconstructed susceptibility distributions.

A strong enforcement of conforming to measurements may degrade image reconstruction quality due to overfitting, particularly if measurements contain noise, errors, or artifacts, such as low SNR, fast signal dropout, and motion artifacts in the case of ICH‐QSM scans. Conversely, placing excessive reliance on generative models while relaxing data consistency risks producing realistic‐looking images that inaccurately represent the underlying scanned objects and lead to hallucination. While our reconstruction parameters were optimized for the high‐susceptibility range of ICH lesions, this choice involves an inherent trade‐off. Prioritizing the suppression of large‐scale artifacts can lead to subtle variations in finer structures. This highlights that hyperparameter tuning in single‐orientation QSM may need to be task‐specific depending on the clinical focus. The accurate guidance of conditional sampling remains an active area of research. Nevertheless, in this ICH‐QSM study, where the primary focus is on lesions that are typically substantial in size and susceptibility value, QSMDiff demonstrates the capability to produce reliable predictions. The other limitation is the relatively slow reconstruction speed (∼15 min) due to the many sampling steps in the reverse diffusion process. Accelerating diffusion models sampling [71, 72] is another active research area, which can benefit QSMDiff in the future.

## 5 Conclusion

This study presents QSMDiff, a new method for QSM dipole inversion using diffusion models, demonstrating robust performance in challenging scenarios. QSMDiff achieves robust and accurate QSM reconstruction for both conventional 3D GRE data and accelerated 2D single‐shot EPI scans in ICH imaging. Experimental results on QSMDiff show strong consistency between ICH‐QSM derived from EPI and SWI, with reduced noise, improved resolution and tissue contrast, and minimized motion and susceptibility artifacts. Its unsupervised, zero‐shot capability enhances its adaptability to diverse QSM acquisition sequences and parameters. QSMDiff establishes the feasibility of fast, accurate QSM evaluation for ICH, showing potential for future clinical translation.

## Funding

This work was supported by the National Health and Medical Research Council (2030157); National Natural Science Foundation of China (62301616); Australian Research Council (DE20101297, DP230101628); Natural Science Foundation of Hunan Province (grant number 2024JJ6530).

## Supporting information


**Figure S1.** Reconstruction results from two simulated acquisition orientations. $\vec{p}$ denotes the orientation vectors and cubes denote for acquisition orientations.
**Figure S2.** QSM reconstruction and super‐resolution test. QSM images were reconstructed from local field maps of varying low resolutions. QSMDiff was compared with iLSQR followed by Sinc interpolation. HR denotes high‐resolution ground truth.
**Figure S3.** Ablation studies on in vivo ICH patients. (a) Subject #3 and (b) Subject #4. Red arrows highlight blurriness or hallucinations observed in the 2D reconstructions.

## Data Availability

The data that support the findings of this study are available on request from the corresponding author. The data are not publicly available due to privacy or ethical restrictions.
