# Supplementary material for: Advancing Quantitative Susceptibility Mapping With 2.5D Diffusion Models for Rapid Intracranial Hemorrhage Quantification
Source: Magn Reson Med. 2026 Mar 24;96(2):596–610. doi: 10.1002/mrm.70358 (PMC13269196; doi:10.1002/mrm.70358)
Supplement: Supplementary file 1 — Figure S1. Reconstruction results from two simulated acquisition orientations. p→ denotes the orientation vectors and cubes denote for acquisition orientations. Figure S2. QSM reconstruction and super‐resolution test. QSM images were reconstructed from local field maps of varying low resolutions. QSMDiff was compared with iLSQR followed by Sinc interpolation. HR denotes high‐resolution ground truth. Figure S3. Ablation studies on in vivo ICH patients. (a) Subject #3 and (b) Subject #4. Red arrows highlight blurriness or hallucinations observed in the 2D reconstructions. [file MRM-96-596-s001.docx]

**Supplementary Material**


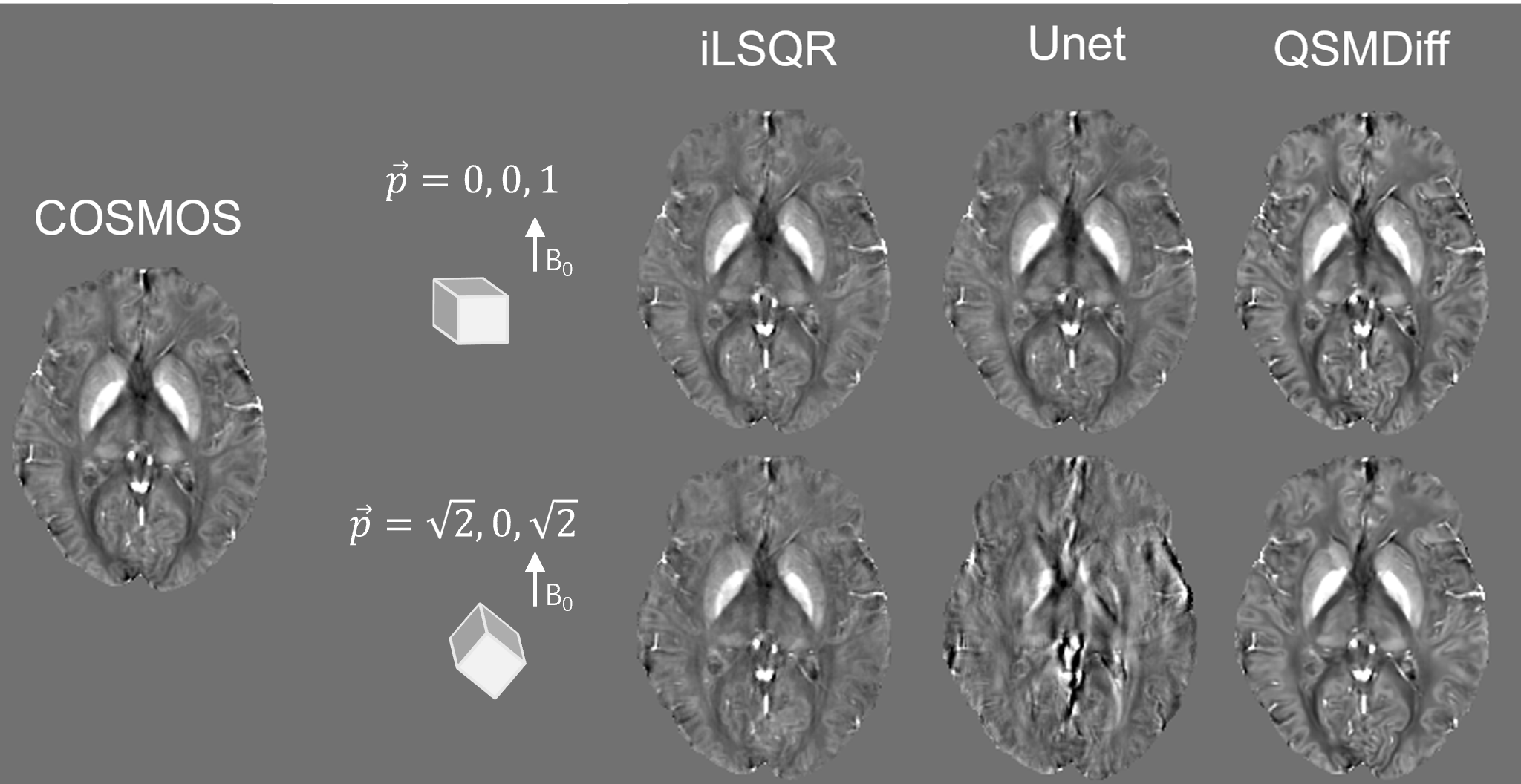


**Figure S1**. Reconstruction results from two simulated acquisition orientations. $\vec{p}$ denotes the orientation vectors and cubes denote for acquisition orientations.


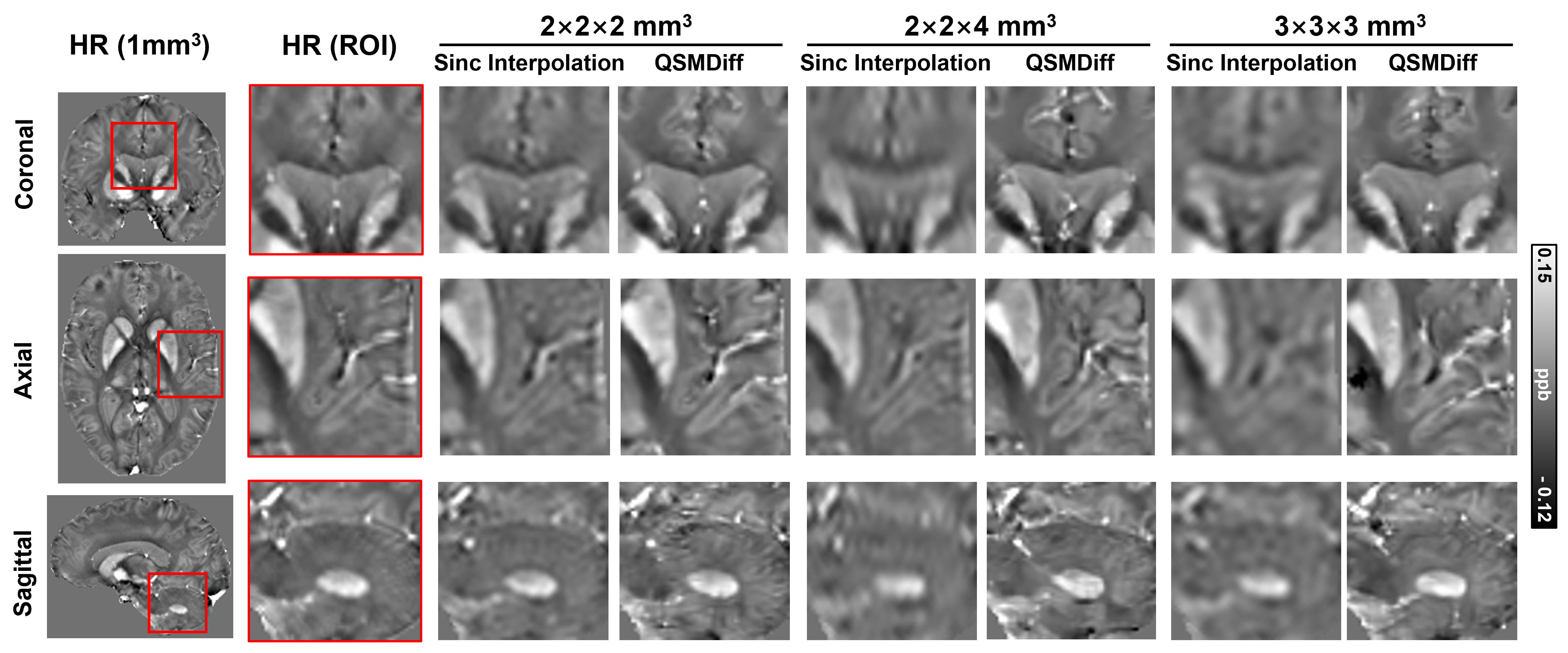


**Figure S2**. QSM reconstruction and super-resolution test. QSM images were reconstructed from local field maps of varying low resolutions. QSMDiff was compared with iLSQR followed by Sinc interpolation. HR denotes high-resolution ground truth.


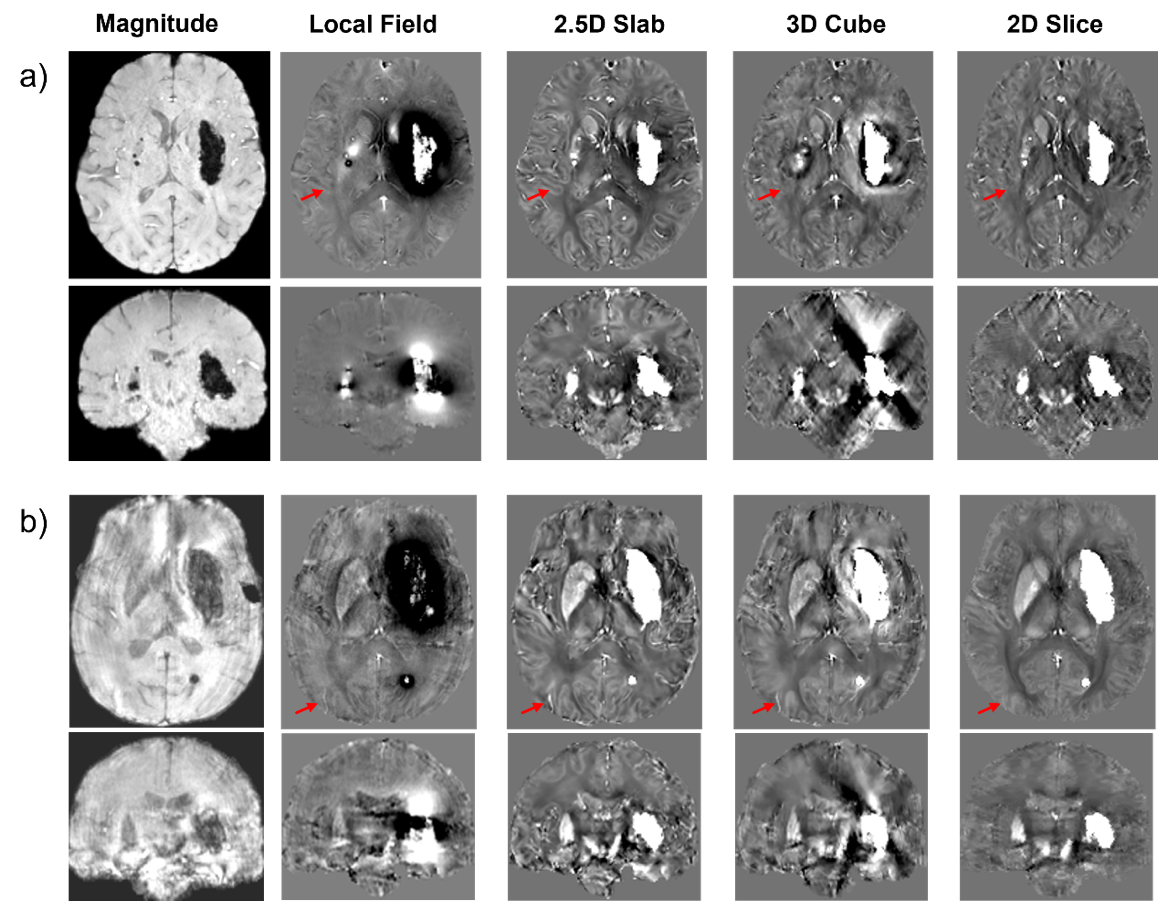


**Figure S3**. Ablation studies on in-vivo ICH patients. (a) Subject #3 and (b) Subject #4. Red arrows highlight blurriness or hallucinations observed in the 2D reconstructions.
